# 1027. At-Home vs. In-Clinic Receipt of Cabotegravir and Rilpivirine Long-Acting: An Implementation Science Trial

**DOI:** 10.1093/ofid/ofad500.058

**Published:** 2023-11-27

**Authors:** Jamila K Williams, Christina Young, Rochelle F Hanson, Angela Moreland-Johnson, Ruth O Adekunle, Stephanie Kirk, Eric G Meissner

**Affiliations:** Medical University of South Carolina, Charleston, South Carolina; Medical University of South Carolina, Charleston, South Carolina; Medical University of South Carolina, Charleston, South Carolina; Medical University of South Carolina, Charleston, South Carolina; Medical University of South Carolina, Charleston, South Carolina; Medical University of South Carolina, Charleston, South Carolina; Medical University of South Carolina, Charleston, South Carolina

## Abstract

**Background:**

Cabotegravir plus rilpivirine long acting (CAB + RPV LA) is administered as an intramuscular injection every 1 or 2 months for treatment of HIV-1 infection. The need for frequent travel to a clinic could impair treatment access. We hypothesized that receiving treatment at-home would be as safe and effective as receiving treatment in clinic and that having different options for where to receive treatment would result in high patient satisfaction.

**Methods:**

Persons prescribed CAB + RPV LA in the Infectious Diseases Clinic at the Medical University of South Carolina (MUSC) were offered enrollment in this implementation science study between August 2021 and December 2022. This study was approved by the MUSC IRB. After receiving the initial injection in clinic, study participants chose to receive each subsequent injection over the 12-month intervention in clinic or at home, with option to switch during the study. For home injections, CAB + RPV LA was shipped to participants’ homes and stored refrigerated until preparation and administration by a home health provider. For clinic injections, CAB + RPV LA was shipped to the clinic and stored until the injection visit. Monthly surveys were completed by study participants.

**Results:**

The thirty-three patients enrolled in the study were primarily Black (64%) and male (73%). Two participants stopped CAB + RPV LA and transitioned to oral therapy due to allergy (n=1) and efficacy (n=1) concerns. Nearly an equal number of participants elected to receive treatment primarily in clinic (n=16) relative to at home (n=14). Contrary to our expectations, switching the primary location of treatment receipt during the study was uncommon (n=2 to date). Most survey responses to date indicated extreme satisfaction (93%) with treatment. Consistent with reported clinical experience, the most common side effect has been injection site pain/soreness (52% of injections). No differences in safety, efficacy, or satisfaction have been observed based on treatment location with results accruing.

**Conclusion:**

Administering CAB + RPV LA at home is associated with high satisfaction and thus far is safe and effective.

**Disclosures:**

**Jamila K. Williams, MHA**, Viiv Healthcare: Grant/Research Support **Stephanie Kirk, PharmD**, Viiv Healthcare: Advisor/Consultant|Viiv Healthcare: Grant/Research Support **Eric G. Meissner, MD, PhD**, Viiv Healthcare: Advisor/Consultant|Viiv Healthcare: Grant/Research Support

